# Global Childhood Deaths From Pertussis: A Historical Review

**DOI:** 10.1093/cid/ciw529

**Published:** 2016-11-02

**Authors:** Maria Yui Kwan Chow, Gulam Khandaker, Peter McIntyre

**Affiliations:** 1 Sydney Medical School; 2 Marie Bashir Institute for Infectious Diseases and Biosecurity Institute, University of Sydney, New South Wales, Australia; 3 Asian Institute of Disability and Development, University of South Asia, Dhaka, Bangladesh; 4 National Centre for Immunisation Research and Surveillance of Vaccine Preventable Diseases, The Children's Hospital at Westmead,New South Wales, Australia

**Keywords:** pertussis, childhood deaths, review

## Abstract

Impact of pertussis vaccines on mortality is a key World Health Organization indicator, and trends in mortality rates and age distribution can inform maternal immunization strategies. We systematically reviewed studies reporting pertussis mortality rates (PMRs) per million population, identifying 19 eligible studies. During a prevaccine observation period of ≥50 years in high-income countries (HICs), PMRs reduced in both infants and 1- to 4-year-olds by >80%, along with improvements in living conditions. In studies in low- and middle-income countries (LMICs), PMRs resembled highest prevaccine HIC rates. Postvaccine in HICs, significant further reduction in deaths (>98%) occurred, but with a large left shift in age of onset among residual deaths. Postvaccine in LMICs, limited data also show large and rapid decreases in PMRs, first in older infants and children, but long-term data fully enumerating residual deaths are lacking. In Sweden, large increases in the prevalence of undetectable pertussis antibodies were found at 10 years after high childhood coverage of acellular pertussis vaccines. Such data are not available from LMICs using whole-cell vaccines in a primary schedule without boosters. Data on residual infant deaths and maternal seroprevalence would be valuable inputs into consideration of pertussis vaccination in pregnancy in LMIC settings, especially if more precise immune correlates of infant protection against death from pertussis were known.

The clinical syndrome of whooping cough has been recognized for at least 500 years, although the causative organism, *Bordetella pertussis*, was not identified until 1906 [1]. As physician diagnosis of the clinical syndrome of whooping cough, in its typical form in unimmunized children, is highly specific when judged against culture as gold standard [2] and an episode of whooping cough is reliably recalled by mothers, even in resource-poor settings [3], historical data on trends in whooping cough incidence should have acceptable validity, if reporting and recording have remained consistent over time.

Similarly, interpretation of historical data on deaths due to pertussis assumes that recognition of the preceding clinical syndrome and recording of deaths has been consistent over time. In countries with good infrastructure, these data come from statistical reports derived from statutory death certificates. More recently, measurement of pertussis-related deaths has been supplemented by data on mortality from routine public health surveillance, often including laboratory confirmation, and capture-recapture methodology has suggested significant underascertainment in both the United States [4] and the United Kingdom [5]. In resource-poor settings, attribution of deaths following clinically diagnosed pertussis may be problematic, especially when death occurs some time later, as other factors such as concomitant infection (eg, malaria, measles), and comorbidities (eg, malnutrition) are often present [3, 6, 7]. Attribution is much clearer when it is in the context of well-established surveillance with clear diagnostic criteria, supplemented by laboratory diagnostic tests, as occurred in study sites in Senegal [8] and Kenya [3, 9] before and after vaccine introduction. However, population-based studies of severe morbidity from pertussis in resource-poor settings are sparse, especially several years after achievement of acceptable vaccine coverage through the Expanded Programme on Immunization (EPI). Recognizing the limitations of the available data sources, deaths due to pertussis have been estimated by modeling, using age-specific estimates of cases, case fatality rate, vaccine coverage, and vaccine effectiveness as the main parameters. In 1999, these methods estimated 295 000 deaths worldwide (52% in Africa and South Asia), but with substantial uncertainty in sensitivity analyses [10]. In 2013, the Global Burden of Disease Study estimated mortality due to pertussis in the first year of life to be approximately 400 per million live births, or approximately 56 000 deaths [11], whereas average annual pertussis mortality rates per 1 million births supplied by 15 (mainly high-income) countries to the World Health Organization (WHO) for the decade 2003–2012 ranged from 0.1 to 38.6, with most between 3.0 and 10.0 [12].

In this review, we aimed to inform considerations for maternal pertussis immunization strategies, and provide historical context, by collating estimates of pertussis mortality rates from population-level studies reporting pertussis deaths in the pre- and postvaccine eras.

## METHODS

### Search Strategy

We searched OVID Medline, OVID Embase, Web of Science, and the Cochrane Library. To maximize retrieval, a combination of database-specific controlled vocabulary terms (where available) and text-word terms were used including the below examples from OVID Medline: *Bordetella pertussis*, *whooping cough*, *pertussis*, *epidemiology*, *incidence*, *prevalence*, *disease notification*, *hospitalization*, *death*, *mortality*, *immunization*, *pertussis vaccine*. To minimize the introduction of bias, no language or date limits were applied. Because no date limits were applied, pertussis case definitions of included studies were either clinically or laboratory confirmed according to the WHO definition.

The search results were exported to an EndNote reference database for screening by one researcher (M. Y. K. C.). Article abstracts or the titles (if abstract was unavailable) were screened, and those deemed likely to provide useful information identified. Full text of articles were retrieved, reviewed, and selected, according to the following criteria:

#### Inclusion criteria

- Any published journal articles that report pertussis deaths or mortality rate, in any language and any time in both pre- and postvaccine era.

#### Exclusion criteria

- Articles that are commentaries, pertussis vaccine recommendations, or case studies;
- Articles that report outcomes only on immune response or reactions after vaccination, or microbiology (eg, strains of bacteria);
- Animal studies;
- Articles that report only on vaccination program delivery and implementation issues and outcomes;
- Articles that report only on pertussis vaccination coverage;
- Articles that report only pertussis vaccination acceptance or attitudes;
- Articles that report only on results of mathematical modeling of pertussis epidemiology;
- Articles that report only on pertussis or vaccine economic evaluation, or cost-effectiveness studies;
- Studies that only or primarily report on estimates of vaccine effectiveness;
- Clinical trials reporting on the efficacy, safety, tolerability, and immunogenicity of pertussis vaccines; and
- Articles that report only on detection of pertussis or surveillance strategies.

References of identified reviews relevant to the topic were screened to identify potentially relevant studies. Articles considered equivocal for inclusion were rescreened by other authors.

### Data Extraction and Outcomes

We extracted data using a standardized data extraction sheet, which included information about the study setting, time and duration of the study, vaccine type, schedule and coverage, year when vaccination program was introduced, ascertainment and definition of deaths, age at death, and denominator population or age-specific incidence.

## RESULTS

We identified 9747 potentially relevant records from the databases used, reduced to 5367 after excluding duplicates, and to 248 after screening titles and abstracts. We further excluded 236 records, which did not provide or allow calculation of population-based mortality rates, leaving 12 studies. Reference search (snowballing) identified 7 additional studies that fulfilled our inclusion criteria. Supplementary Figure 1 shows the process of screening for relevant studies.

### Characteristics of Eligible Studies

The included studies were published between 1945 and 2014, and provided mortality data from 1860 to 2012. For high-income countries (HICs), studies were from Argentina [13], Australia [14], Denmark [15, 16], Germany [17], Ireland [18], Italy [19], New Zealand [20], Switzerland [21], the United Kingdom [22–23], and the United States [24–26]. For low- and middle-income countries (LMICs), studies were from Kenya [3, 9], Senegal [8], South Africa [27], and Turkey [28]. From HICs, all studies were summary descriptive accounts of administrative data, in most exclusively from statutory death certificates. One study from Denmark used death records from a single hospital [15] and 1 study from the United Kingdom employed capture-recapture methods from 3 data sources [22]. Among the 5 eligible studies from 4 LMICs [3, 8, 9, 27, 28], 2 were community-based prospective cohorts in Senegal [8] and Kenya [3, 9].

### Pertussis Mortality Rates Prior to Vaccine Introduction

Table 1 shows studies that provided data on pertussis mortality rates before vaccine introduction, either for all ages or for specific age groups, expressed as a rate per million of the relevant population denominator.

**Table 1.** Pertussis Mortality Rates in the Prevaccine Era

Country	Data Sources	Vaccine Schedule Introduced	Study Period	Age Group	Mortality per Million Population
Low- and middle-income countries					
Kenya [3, 9]	Prospective cohort Household survey	1980s	1974–1976 and 1974–1977	All ages <1 y 1 to <4 y ≥5 y	480 (95% CI, 410–570) 5110 (95% CI, 4090–7160) ∼1800 20
Senegal [8]	Prospective cohort Verbal autopsy	1986–1987	1986	0–5 mo 6–23 mo 2–4 y 5–14 y <15 y	6900 6200 4000 200 2200
South Africa [27]	Statutory death certification (national)	1974	1939–1947	All ages	European: 30 Native: 120
High-income countries					
Australia [14]	Statutory death certification (national)	Introduced: 1940s Widely used: 1950s	1936–1945	All ages	235
Denmark [15]	Death records (single fever hospital ∼40% of identified discharges)	1960s	1919–1935	All ages <1 y	51 1712
Denmark^a^ [16]	Statutory death certification (national)	1960s	1856–1930	All ages	1860: 1500 1930: 400
Germany [17]	Statutory death certification (national)	1962	1959	<1 y	343
Ireland [18]	Statutory death certification (national)	Mid 1950s	1940–1949; 1950–1954	All ages	67; 23.9
Italy [19]	Statutory death certification (national)	1961	1890 and 1960	All ages	1890: 425 1960: 2
New Zealand [20]	Statutory death certification (national)	1960	1950–1959	All ages	7.6
Switzerland [21]	Unspecified	1950	1931–1950	All ages	20.7
UK [23]	Statutory death certification (national)	1950s	1944–1949; 1950–1957	<1 y 1–4 y 5–9 y	709^b^; 207^c^ 108^b^; 22^c^ 6.6^b^; 1.3^c^
US [25]	Statutory death certification (national)	Introduced: 1940s Widely used: 1950s	1900–1919; 1920–1934; 1934–1949	<1 y 1–4 y	3530^d^; 2743^e^; 1040^f^ 653^d^; 797^e^; 128^f^
US^g^ [24]	Statutory death certification (national)	Introduced: 1940s Widely used: 1950s	1938–1940	All ages <1 y	27 1176

Abbreviations: CI, confidence interval; UK, United Kingdom; US, United States.

^a^ Reported by Wright [16]; derived from: Madsen T. Lectures on the epidemiology and control of syphilis, tuberculosis, whooping cough, and other aspects of infectious disease. Baltimore: Williams & Wilkins, 1937.

^b^ Average of figures for 1944–1945 and 1946–1949.

^c^ Average of figures for 1950–1953 and 1954–1957.

^d^ Average of figures for 1900–1919.

^e^ Average of figures for 1920–1934.

^f^ Average of figures for 1934–1939.

^g^ Only number of deaths was given; we calculated the incidence according to population data.

#### Mortality Rates—All Ages

**Figure 1.**
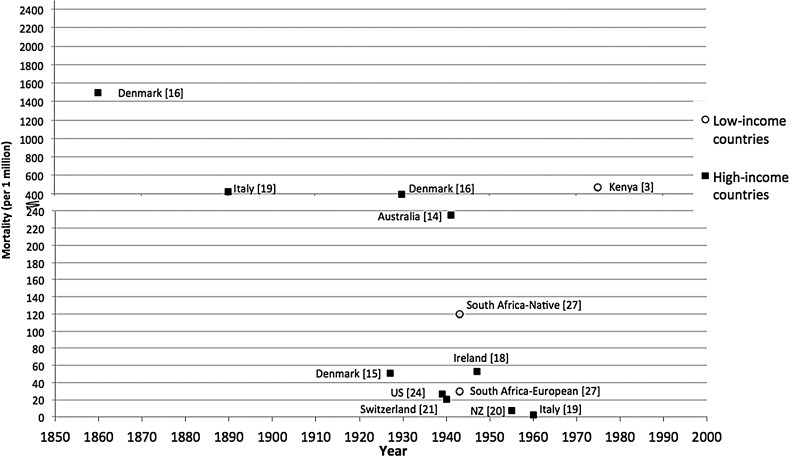
All-age pertussis mortality in the prevaccination era. Voorhoeve et al [3]: 1974–1977 (N = 12), mortality rate = 470 per million. Brotherton et al [14]: 1936–1945 (N = 1693), mortality rate = 235 per million. Nielsen et al [15]: 1919–1935 (N = 482), mortality rate = 51 per million. Wright [16]: 1860 (N = unknown), mortality rate = 1500 per million; 1930 (N = unknown), mortality rate = 400 per million. Howell and Jennings [18]: 1940–1944 (N = 58), mortality rate = 73.8 per million; 1950–1954 (N = 66), mortality rate = 23.9 per million. Gonfiantini et al [19]: 1890 and 1960 (N = unknown), mortality rate = 425 and 2 per million; Somerville et al [20] 1950–1954 (N = 119), mortality rate = 11.9 per million; 1955–1959 (N = 37), mortality rate = 3.3 per million. Wymann et al [21]: 1931–1950 (N = 91) (annual average), mortality rate = 20.7 per million. Sako et al [24]: 1938–1940 (N = 10 730), mortality rate = 27 per million. Ordman [27]: 1939–1947 (native N = 80), mortality rate = 120 per million; (European N = 278), mortality rate = 30 per million. Abbreviation: NZ, New Zealand.

All-age pertussis mortality rates (per million population) in industrialized countries declined from the 19th to the early 20th century (1500 in Demark in 1860 [16]; 425 in Italy in 1890 [19], 400 in Denmark in 1930 [16], 235 in Australia in 1936–1945 [14]), at which time they were comparable to mortality rates reported from Kenya in the 1970s [3, 9]. These trends over time are displayed graphically in Figure 1, which demonstrates substantial divergence in reported rates across industrialized countries from the 1930s.

#### Mortality Rates—Infants and Children

**Figure 2.**
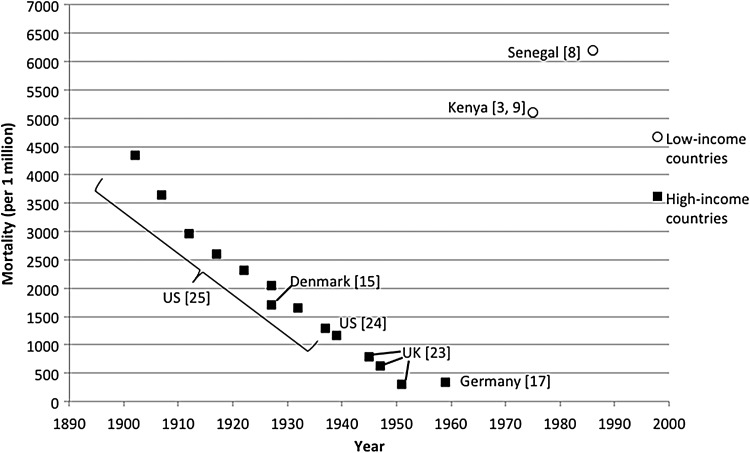
Pertussis mortality in infants <1 year of age in the prevaccination era. Mahieu et al [9] and Voorhoeve et al [3]: 1974–1976 (N = 5), mortality rate = 5110 per million. Preziozi et al [8]: 1986 (N = 13), mortality rate = 6200 per million (ages 0–23 months). Nielsen et al [15]: 1919–1935 (N = 291), mortality rate = 1712 per million. Mebel and Dittmann [17]: 1959 (N = unknown), mortality rate = 343 per million. Amirthalingam et al [23]: 1944–1945, mortality rate = 796.3 per million; 1946–1949, mortality rate = 620.6 per million; 1950–1953, mortality rate = 307.7 (all N unknown). Sako et al [24]: 1938–1940 (N = 7123), mortality rate = 1176 per million. Mortimer and Jones [25]: 1900–1904, mortality rate = 4340 per million; 1905–1909, 3650 per million; 1910–1914, 2960 per million; 1915–1919, 2600 per million; 1920–1924, 2310 per million; 1925–1929, 2050 per million; 1930–1934, 1660 per million; 1935–1939, 1300 per million (all N unknown).

Similar to current reports, historical mortality rates from pertussis in infancy were higher than at any other age, but declined progressively over time during the prevaccine era. In the United States, the mortality rate per million births in the first year of life declined by about 70% over the first half of the 20th century (3530 in 1900–1919 vs 1040 in 1934–1949) [25], with Sako et al reporting a similar mortality rate in this age group between 1938 and 1940 [24]. These trends are displayed graphically in Figure 2, which demonstrates an apparent linear decline in infant mortality in HICs during the prevaccine period. In contrast, the reported infant mortality rate from pertussis in the 1970s and 1980s from Kenya (5110) [3, 9] and Senegal (approximately 6200) [8] was about 2-fold higher than the United States before 1920, prior to any declines in mortality rates in that country [25].

The mortality rate among children aged 1–4 years in the United States declined more steeply (84%) than in infants (797 in 1900–1919 vs 128 in 1934–1939) [25], remaining around one-tenth of that in infants. In the United Kingdom, the mortality rate among 1- to 4-year-olds declined by 80%, from 108 per million in 1944–1949 to 22 per million in the period immediately before vaccine was widely used (1950–1957) [23]. In Kenya and Senegal in the 1970s, mortality rates in children aged 1 to <5 years were between 3- and 6-fold higher than in the United States prior to 1920 (Table 1).

### Reported Pertussis Mortality Rates Following Vaccine Introduction

Table 2 shows studies with data following vaccine introduction in LMICs and HICs, with study periods from the 1950s onward. All-age mortality rates in HICs ranged from 3.4 to 5.3 per million in the early postvaccine period (Australia [14], Ireland [18], and Italy [19]), decreasing by approximately 90% in later periods [14, 18], while Argentina reported a rate of 1.9 for 1980–2000 [13]. Infant mortality per million births also decreased from the early postvaccine period (12.3 in the United States in 1964–1974 [25] and 16.5 in the United Kingdom in 1966–1973 [23]) to later (2.4 in the United States in 1990–1999 [26] and 7.2 in the United Kingdom in 2001–2011 [22]). The only low-income country reporting mortality after vaccine introduction was Senegal, which had a profound decrease in identified pertussis deaths in children aged >2 years to zero within 3 years of vaccine introduction, but no substantive decrease in this time frame among those aged <2 years (6200 per million [8]). However, at 6 years after introduction, identified pertussis mortality among infants 0–5 months of age had decreased from 6900 to 1300 per million [8].

**Table 2:** Pertussis Mortality Rates in the Postvaccine Era

Country	Type of Study and Data Source	Vaccine Schedule Introduced	Study Period	Age Group	Mortality per Million Population
Low- and middle-income countries					
Senegal [8]	Prospective cohort Household interview and diagnosis by experienced clinicians	1986–1987	1990; 1993	0–5 mo 6–23 mo 2–14 y <15 y	6900; 1300 6200 0.0 600
Turkey [28]	Statutory death certification (national)	1985	1986–2005	All ages	0
High-income countries					
Argentina [13]	Statutory death certification (national)	1970s	1980–2000	All ages <1 y	1.9 65.6
Australia [14]	Statutory death certification (national)	Introduced: 1940s Widely used: 1950s	1956–1965; 1966–1995; 1996–2005	All ages	5.3^a^; 1.1^b^; 0.36^c^
Germany [17]	Statutory death certification (national)	1962	1965–1970	All ages	0.2
Ireland [18]	Statutory death certification (national)	Mid 1950s	1955–1969^a^; 1970–1984^b^	All ages	4.2^d^; 0.3^e^
Italy [19]	Statutory death certification (national)	1961	1961–1994	All ages	1961: 3.4 1994: <10
New Zealand [20]	Statutory death certification (national)	1960	1960–2004	All ages	0.4
Switzerland [21]	National surveillance + case definition	1950	1971–1990	All ages	0.92
UK [23]	Statutory death certification (national)	1950s	1958–1965; 1966–1973	<1 y 1–4 y 5–9 y	27.3^f^; 16.5^g^ 2.5^f^; 0.35^g^ 0.2^f^; 0.05^g^
UK [22]	Statutory death certification (national) and notification data (capture/recapture)	1950s	2001–2011	<1 y	7.21 (95% CI, 7.05–7.63)
US [25]	Statutory death certification (national)	Introduced: 1940s Widely used: 1950s	1950–1959; 1960–1974	<1 y	85^h^; 12.3^i^
US [26]	Statutory death certification (national) and notified cases known to have died	Introduced: 1940s Widely used: 1950s	1990–1999	<1 y	<1 y: 2.40 (95% CI, 1.95–2.92)

Abbreviations: CI, confidence interval; UK, United Kingdom; US, United States.

^a^ Average of figures for 1956–1965.

^b^ Average of figures for 1966–1975, 1976–1985, and 1986–1995.

^c^ Average of figures for 1996–2005.

^d^ Average of figures for 1955–1959, 1960–1964, and 1965–1969.

^e^ Average of figures for 1970–1974, 1975–1979, and 1980–1984.

^f^ Average of figures for 1958–1961 and 1962–1965.

^g^ Average of figures for 1966–1969 and 1970–1973.

^h^ Average of figures for 1950–1954 and 1955–1959.

^i^ Average of figures for 1960–1964, 1965–1969, and 1970–1974.

### Age Distribution of Pertussis Deaths

#### Prevaccine Period—United States Versus Nigeria and Senegal

**Figure 3.**
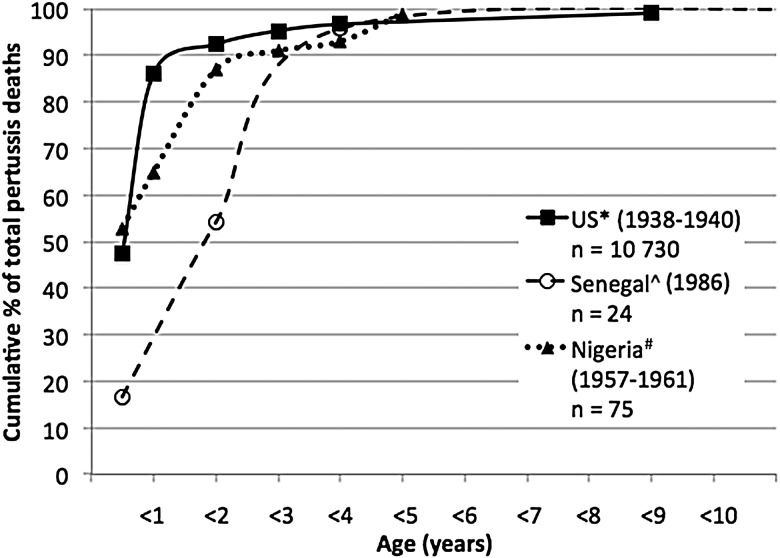
Age distribution of pertussis deaths in the United States (US) and Senegal in the prevaccine era. *Sako et al [24]; ^Preziosi et al [8]; ^#^Morley et al [6].

Detailed data on the age distribution of pertussis deaths in the prevaccine era are available from only 1 US study reporting 10 730 cases in 1938–1940 [24]. Data from 2 much smaller prevaccine studies in Africa (Nigeria [6] and Senegal [8]) are contrasted with the United States in Figure 3. The distribution of cases <5 years of age appears to be similar in the US and Nigerian studies (both of which can be assumed to be hospital-based), but a smaller proportion of cases in the first 6 months of life was identified in the study from Senegal, which was community-based.

#### Prevaccine Period Versus Postvaccine Period—United States

A recent US study provides detailed age distribution by week of age for 258 deaths identified through national case surveillance data rather than death certificates between 1991 and 2008 [29]. In the prevaccine period, 67% of deaths occurred by 12 months of age, but <5% were identified in the first month of life and around 15% by 2 months [24], in contrast to the US experience in 1991–2008, where almost 50% of deaths had occurred by age 1 month and 85% by 2 months [29].

## DISCUSSION

There are significant limitations in the nature and quality of the data available on pertussis mortality derived from statutory death certificates and presented in secondary reports of the primary data over an extended period, as is the case in studies from the United States [16, 25], the United Kingdom [23], and Australia [14]. Overall, it is reasonable to assume that the pertussis mortality rates derived from these studies represent minimum estimates; their approximate concordance by setting and historical period is reassuring and allows some broad conclusions.

First, the data presented here serve to remind us that childhood mortality from pertussis in the prevaccine era was substantial, in both HICs and LMICs. A report from the United States noted that between 1940 and 1944, whooping cough accounted for more than twice as many childhood deaths as the combined total for measles, scarlet fever, diphtheria, polio, and meningococcal infections [30]. In the Netherlands, a more sophisticated statistical analysis has estimated that pertussis accounted for 3.7% of deaths before the age of 20 years between 1903 and 1946, decreasing to 0.024% after vaccine introduction in 1954 [31]. In Kenya, pertussis was estimated to have contributed to 4% of all deaths in children aged <15 years [3] and in Senegal to 9% of deaths in children aged <5 years [8].

Second, childhood deaths extended well beyond infancy. A detailed estimate from a vaccine trial in a small rural settlement in Kenya found that the total number of deaths (7) identified in children between the ages of 1 and 5 years was higher than that identified in children <1 year of age (5) and that this represented approximately 5% of all deaths in children <2 years of age but >10% of deaths in children between 2 and 5 years of age [9].

Third, in HICs, deaths from pertussis decreased substantially over the 100 years prior to vaccine availability, which at earliest was from the 1940s, presumably due to improvements in both living conditions and medical care. Morley quoted a thesis undertaken in Aberdeen, Scotland, on pertussis deaths in the late 19th century, which identified that pertussis deaths were significantly associated with both the total number of children and the number of rooms in households [6]. The data available from high-mortality countries in Africa (Kenya and Senegal) show that pertussis mortality burden in the 1970s and 1980s in these countries was of a similar order to that identified in the late 19th and early 20th centuries in Denmark and the United States [16]. In settings where infant mortality is high, pertussis vaccine coverage very low, and access to health services limited, pertussis mortality in intense epidemics can be very high even in contemporary times [32].

Fourth, vaccine introduction led to further large and rapid reductions in pertussis deaths. In the Netherlands, pertussis mortality and population data from the 1920s were used to demonstrate that, after accounting for reductions in mortality rates occurring prior to vaccine introduction, reductions observed after vaccine introduction remained highly statistically significant [31]. The early impact of introduction of pertussis vaccines documented in Kenya and Senegal was rapid and substantial, even prior to reaching high coverage in relevant birth cohorts, and was associated with herd effects [8, 9]. With respect to deaths, it is notable that the first and most dramatic reduction was for deaths in older children, who would have had the most robust immune responses to pertussis vaccine, rather than infants [8]. In The Gambia, pertussis epidemics were described as affecting more than two-thirds of children <5 years of age in a detailed study at the village level in 1962 [7]; by the 1990s, when high pertussis vaccine coverage had been in place for >10 years, severe pertussis was reported as being “almost unknown” [33].

**Figure 4.**
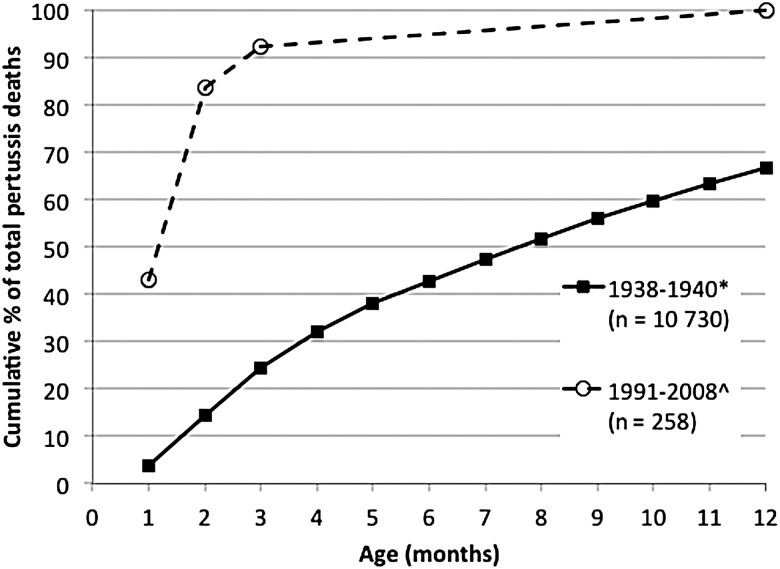
Age distribution of pertussis deaths in children <12 months of age in the United States in the pre- and postvaccine eras. *Sako et al [24]; ^Tiwari et al [29].

Shortly after the introduction of pertussis vaccines into infant and later childhood programs, concern was raised that reduction in the intensity of pertussis transmission in children could lead over time to loss of maternal immunity [25, 33]. Although the occurrence of early infant cases of pertussis in the prevaccine era indicated that transfer of maternal immunity was less reliable and/or complete than for measles, where early infant cases are rare unless the mother herself develops measles in late pregnancy, the shifting to the left of the age distribution of deaths from pertussis in countries with long-standing high pertussis vaccine coverage, as strikingly demonstrated for the United States in Figure 4, is suggestive of lesser degrees of infection-acquired maternal immunity over time. The potential contribution of waning maternal immunity after vaccine introduction has been well demonstrated in Sweden, where the proportion of mothers with undetectable antibody to pertussis toxin increased dramatically by 10 years after high coverage with acellular pertussis vaccines in childhood programs with steadily increasing booster doses [34]. However, no such data are available from countries after introduction of whole-cell vaccines, especially when used only for 3 doses in the primary EPI course at 6, 10, and 14 weeks. It is plausible in such settings that continuing high circulation of pertussis in children and adults could boost antibody levels in those primed by whole-cell vaccine or infection, such that relatively high levels of immunity, including maternal immunity, are maintained in older age groups. It is also plausible that difficulties in detecting deaths due to pertussis in early infancy, noted by Morley in the 1960s in an intensely studied Nigerian village to occur unexpectedly in infants with apparently relatively mild symptoms [6], and limited reporting of early infant deaths in such settings, could mask increases in neonatal pertussis deaths. In low-income countries, new studies rigorously assessing the true burden of severe early infant pertussis in settings with differing infant vaccine coverage, as well as data on the immune profile of mothers, would allow more informed assessment of the likely value of maternal pertussis vaccine strategies, especially if studies in HICs where maternal immunization is being practiced enable better delineation of the correlation between maternal antibody at birth and protection against early infant death.

## Supplementary Data

Supplementary materials are available at http://cid.oxfordjournals.org. Consisting of data provided by the author to benefit the reader, the posted materials are not copyedited and are the sole responsibility of the author, so questions or comments should be addressed to the author.

Supplementary Data
