# Supplementary material for: Global Childhood Deaths From Pertussis: A Historical Review
Source: Clin Infect Dis. 2016 Nov 2;63(Suppl 4):S134–41. doi: 10.1093/cid/ciw529 (PMC5106618; doi:10.1093/cid/ciw529)
Supplement: Supplementary Data [file supp_63_suppl-4_S134__index.html]

Supplementary Data 

# Global Childhood Deaths From Pertussis: A Historical Review

## Supplementary Data

Supplementary Data

- Supplementary Data - Pdf file
