# Supplementary figures and images for: Global Childhood Deaths From Pertussis: A Historical Review
Source: Clin Infect Dis. 2016 Nov 2;63(Suppl 4):S134–41. doi: 10.1093/cid/ciw529 (PMC5106618; doi:10.1093/cid/ciw529)

## Supplemental Figure 1

### PRISMA flow chart showing the identification of the studies

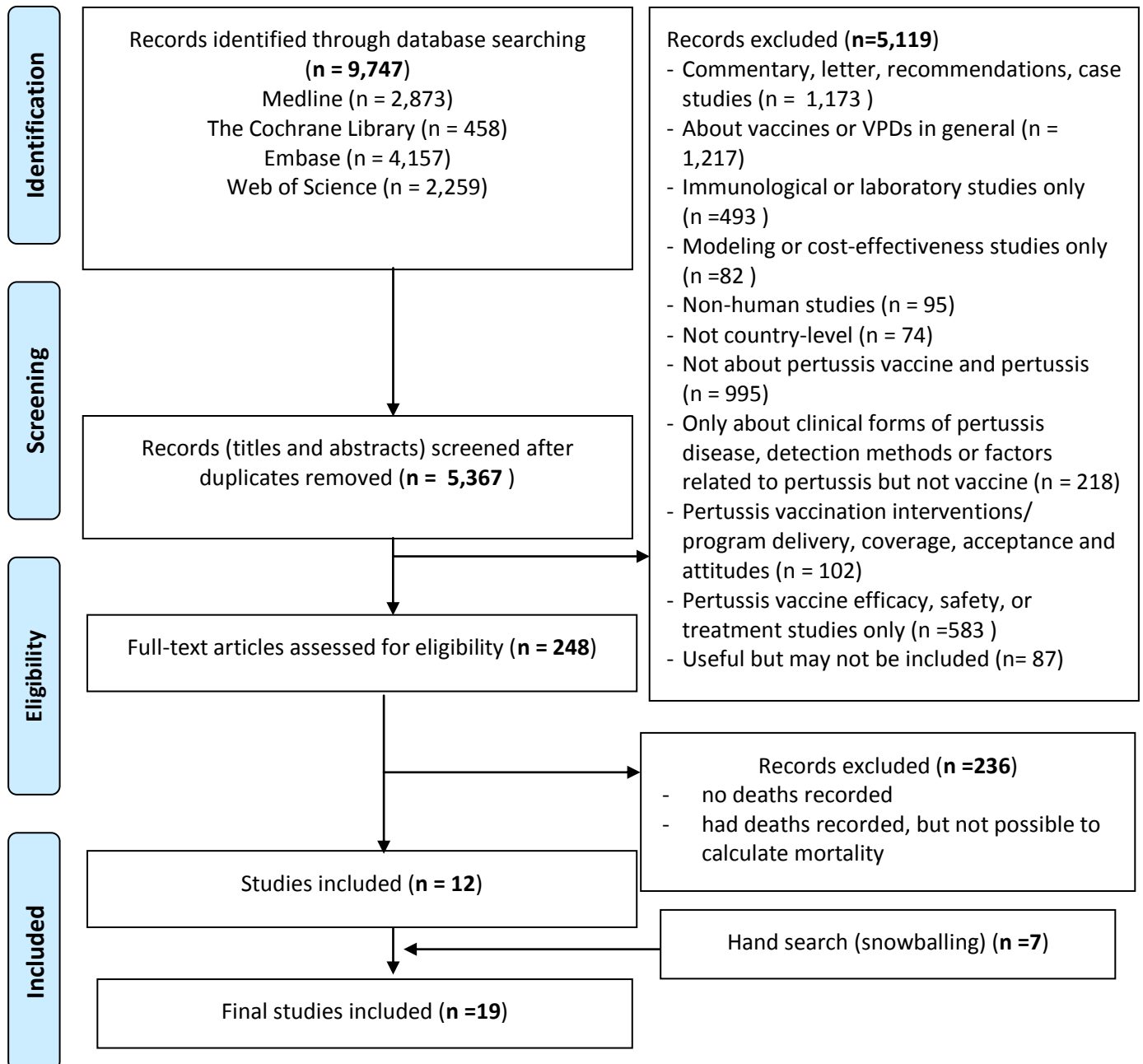

Supplement: Supplementary Data [file supp_ciw529_ciw529supp.pdf]
